# Hyperinosis

**Published:** 1912-11

**Authors:** 


### ABSTRACTS.

---

HYPERINOSIS, the cause of death in a case of chronic parenchymatous nephritis, Dr. Allard Memminger, Charleston, S. C., *St. Louis Med. Rev.*, Sept., 1911. Male, age 23, dropsical. Diagnosis of large, white swollen kidney, verified post mortem. No accumulation in serous cavities. Fibrinous clot in right heart and verna cava ascendens.

Note. It is somewhat questionable whether in such cases hyperinosis may be considered the cause of death or, indeed, whether there is hyperinosis at all. The late H. F. Formad of Philadelphia, used to dwell on the fact that soft, red clots in the heart and great vessels were formed post mortem and occurred in cases in which death occurred rather suddenly while, on the other hand, any case in which the process of dying was prolonged, that is to say in which the ordinary vital processes including the speed and force of the circulation were much reduced for a period of many hours or several days, allowed the formation of fibrinous clots, unless there was a surcharge of carbon dioxid or other condition tending to prevent coagulation. It is also questionable whether the author is right in ascribing dropsy to construction of the vessels with fibrinous clots. It is of interest to note the occurrence of anasarca without cavity dropsy and some of our readers may be able to explain this point. Another point for discussion which must occur to everyone who quantitates albumin in urine, instead of judging by the bulk of the moist coagulum, is as to the actual effect of an albuminuria. In the present case, the loss of albumin was much more than in the average chronic Bright's and rather high for an acute case. Very seldom does the loss amount to 5 grams a day. Very seldom does the urinary loss amount to so much as may be added to the ingesta and as may, apparently, be assimilated.

